# Frequency of Comorbidities in Admitting COVID-19 Pneumonia Patients in a Tertiary Care Setup: An Observational Study

**DOI:** 10.7759/cureus.13546

**Published:** 2021-02-25

**Authors:** Muneer Hussain, Samar Iltaf, Salma Salman, Faiza Ghuman, Saira Abbas, Meraj Fatima

**Affiliations:** 1 Medicine, Dow University of Health Sciences, Karachi, PAK; 2 Neurology, Dow University of Health Sciences, Karachi, PAK

**Keywords:** covid-19 and diabetes, ischemic heart disease, idiopathic hypertension, coronavirus disease 2019, end-stage renal disease (esrd)

## Abstract

Background

The novel coronavirus disease 2019 (COVID-19) is a highly infectious and pandemic disease with a variable mode of action. Patients with underlying illnesses such as diabetes, hypertension, and other diseases are more prone to infection. An understanding of the different comorbidities that place patients at the highest risk of COVID-19 pneumonia and other fatal complications associated with COVID-19 is necessary for healthcare professionals. This study aimed to determine the frequency of different comorbid illnesses among COVID-19 patients admitted to a tertiary care hospital in Karachi, Pakistan.

Methodology

All patients diagnosed with COVID-19 who required admission for the care of their symptoms were included in this observational, cross-sectional study conducted from May 1 to July 30, 2020. The patients were treated at a specialized COVID-19 isolation ward built at the Dow University of Health Sciences at the Ojha campus. The patients were referred from the emergency department, medical and allied wards, and COVID-19 screening units. A detailed history and clinical examination were performed, and comorbidities were evaluated.

Results

A total of 212 patients were admitted during the study with a mean age of 52 ± 16 years. The study population consisted of 120 (56.6%) males and 92 (43.39%) females, and the most common comorbidities were uncontrolled diabetes with hypertension (n = 56; 26.4%), controlled diabetes (n = 22; 10.37%), obstructive airway disease (n = 16; 7.5%), and interstitial lung disease (n = 14; 6.6%). A total of 48 (22.64%) patients had no comorbidities.

Conclusions

Most COVID-19-positive patients with pneumonia were male, and common comorbidities included uncontrolled diabetes, hypertension, and obstructive and restrictive lung disease. The presence of comorbidities was associated with a marked increase in the risk of morbidity and mortality. Further studies are warranted to confirm these findings.

## Introduction

The outbreak of coronavirus disease 2019 (COVID-19) caused by the severe acute respiratory syndrome coronavirus 2 (SARS-CoV-2) since November 2019 has become a global pandemic, infecting millions of people with a death toll still rising globally. As SARS-CoV-2 begins to evolve, there remain deficits in our understanding of whom this virus will impact. Elderly adults and individuals of any age with chronic medical problems, such as asthma, hypertension, and diabetes, have demonstrated poorer prognosis [1]. At admission, 20% to 51% of patients reported having at least one comorbidity, with diabetes (10% to 20%), hypertension (10% to 15%), and other cardiovascular and cerebrovascular disorders (7% to 40%) being the most prevalent [2-4]. A systematic analysis of middle-aged and elderly patients with COVID-19 showed that the older population is more vulnerable to this condition and more likely to be admitted to intensive care units with higher mortality rates [5]. Despite the high frequency of COVID-19 cases, there is insufficient knowledge about comorbid illness complicating pneumonia in our society.

The purpose of this study was to evaluate different comorbid illnesses in COVID-19 pneumonia in our community. Because of the massive economic, mental, and physical burden on the healthcare system, a thorough assessment and evaluation of all comorbidities and related conditions are needed to fight this deadly pandemic. This study will help us identify vulnerable groups and people at risk, which will ultimately help healthcare workers, scientists, and administrative personnel in developing new recommendations and guidelines to address COVID-19.

## Materials and methods

This observational, retrospective, and descriptive study was performed in a specially designed COVID-19 department at the Dow University Hospital (DUHS), one of the largest tertiary care facilities with COVID-19 isolation units. The study duration was three months from May 1 to July 30, 2020. Participants provided informed written consent, and the ethical committee of DUHS approved the study design.

All patients diagnosed with COVID-19 via pharyngeal or nasopharyngeal swabs with polymerase chain reaction screening positive for SARS-CoV-2 who required hospitalization for their symptoms such as shortness of breath, fever, cough, chest pain, altered sensorium, and anosmia who presented in outpatient and inpatient emergency wards were included in the study. Patients positive for immunoglobulin G antibodies to SARS-CoV-2 were excluded. Patients aged 14 to 80 years of both sexes were included in our study. Patients with underlying diabetes, hypertension, ischemic heart disease, and chronic obstructive pulmonary disease (COPD) were included in our study. The patients were referred from the emergency department, medical and allied wards, and COVID-19 screening units. We reviewed detailed histories, clinical examination findings, and comorbidities of patients.

Along with baseline complete blood workup, we included acute-phase reactants in our study including C-reactive protein, D-dimers, procalcitonin, erythrocyte sedimentation rate, troponin-I, creatine kinase-myocardial band, and glycated hemoglobin (HbA1c) measured in all patients on the first day of admission and during the illness to monitor their response to treatment. Chest X-ray, electrocardiogram, chest computed tomography, and echocardiography were performed in all patients. All data on demography, clinical history, and body mass index were recorded by a principal investigator on a predesigned proforma.

The results were analyzed by SPSS Statistics for Windows, Version 25.0 (IBM Corp., Armonk, NY, USA). Data were presented as frequency and percentages for qualitative variables, while mean and standard deviation were presented for quantitative measures. A Chi-square test was applied, and p ≤ 0.05 was considered a statistically significant difference.

## Results

**Figure 1:**
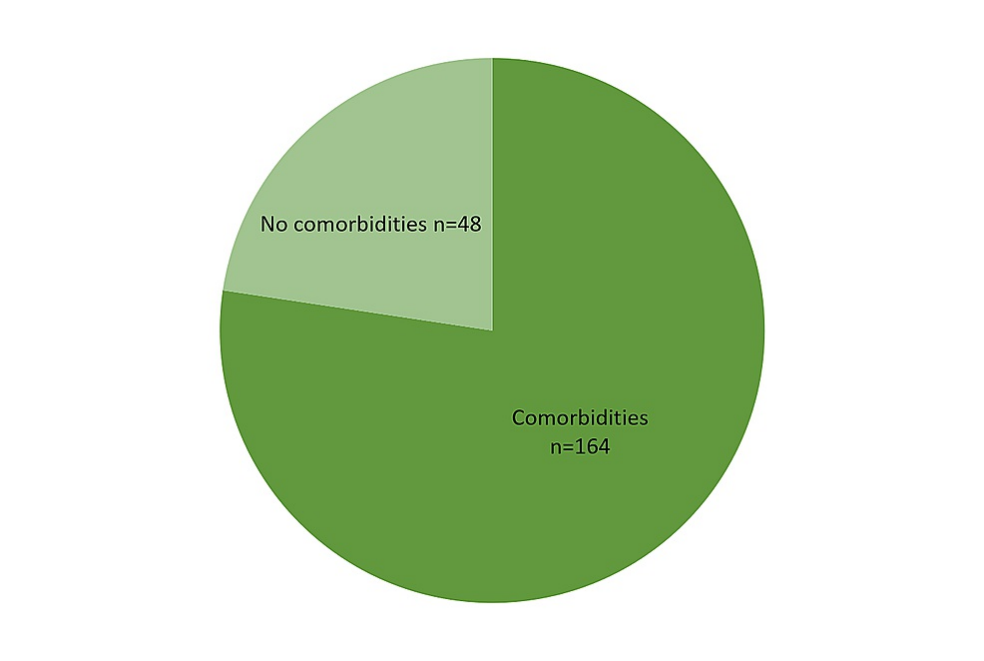
Proportion of patients with comorbidities and patients without comorbidities (N = 212).

A total of 212 patients were included in the study, consisting of 120 (56.6%) male patients and 92 (43.39%) female patients. The mean age of the study population was 52 ± 16 years. A total of 164 patients presented with comorbidities, and 48 patients reported no concomitant comorbid illness (Figure 1). The frequency of different comorbid conditions in COVID-19 pneumonia is shown in Table 1.

We considered the healthy fasting blood glucose levels to be 70-110 mg/dl, and we considered HbA1c below 6.5 as healthy. Patients with controlled diabetes (n = 22; 10.37%) had HbA1c ranging from 6.5 to 7.5, and uncontrolled diabetes was represented by HbA1c >8.5 (n = 56; 26.4%). A total of 16 (7.5%) patients had COPD, six (2.83%) had underlying chronic renal failure, and 48 (22.64%) had no comorbid illnesses (Table 1).

 

**Table 1:** Frequency of comorbid conditions in COVID-19 patients (N = 212). COVID-19, coronavirus disease 2019; DM, diabetes mellitus; HTN, hypertension; IHD, ischemic heart disease; CCF, chronic cardiac failure; ESRD, end-stage renal disease

Comorbidities	Number of cases	Percentage
DM (controlled)	22	10.37%
Uncontrolled DM with HTN	56	26.4%
DM with IHD	10	4.71%
DM with CCF	7	3.3%
Diabetic foot	3	1.41%
ESRD	6	2.83%
Obstructive airway disease	16	7.5%
Interstitial lung disease	14	6.60%
No comorbidities	48	22.64%

## Discussion

Overall, our results contrasted with the previously published research on the commonality of comorbidities in COVID-19 patients. The most prevalent comorbidities associated with worse prognosis have included diabetes [6,7], asthma, respiratory disorders, pulmonary failure [8,9], obesity, kidney disease [8], pregnancy, cardiac diseases, hypertension, and malignancy. Our results indicate that, compared to other significant acute respiratory events, comorbidities such as COPD, asthma, hypertension, and malignancy are predisposed to adverse health effects in COVID-19 patients.

Diabetes was the most common comorbid condition in our study, with 22 (10.37%) patients having controlled their glycemic levels and 56 (26.4 %) with uncontrolled glycemic levels, which was much higher than the incidence of diabetes found in a study conducted in Wuhan, China (10% to 15%) [1-4]. The second most common comorbid condition in our patient population was ischemic heart disease with diabetes (n = 10; 4.71%) and congestive heart failure with diabetes (n = 7; 3.3%). In the study conducted in Wuhan, China, the second most common comorbidity was hypertension (n = 269; 16.9%) and other cardiovascular diseases (n = 59; 3.7%) [2,5,10]. The incidence of obstructive airway disease was lower in our study (n = 16; 7.5%) compared to the study in China (50.0%) [11].

The frequency of the correlation between various comorbidities and the prognosis was less robust according to various studies in the literature [12]. For example, the risk between heart disorders and poor clinical outcomes of influenza, SARS-CoV, or Middle East respiratory syndrome-CoV outbreaks was not definitive. A systematic analysis indicated that SARS-CoV infections contributed to immune repression that may further explain the elevated risk of heart disease, bone disease, and malignancy [13].

COPD [14] and diabetes [15] also coexist with hypertension and ischemic heart disease. Our results indicated that those with comorbidities have higher illness intensity than those without comorbidities. A higher number of comorbidities is associated with a higher disease incidence of COVID-19. Related comorbidities such as asthma, COPD, diabetes, and cardiovascular disease are more important risk factors in patients relative to other underlying conditions [16]. Apart from intensive support initiatives, comprehensive care has yet to be established. Health regulators theorize that individuals with comorbid conditions have a more significant disease consequence when afflicted with COVID-19 than patients with no underlying conditions [17].

## Conclusions

Most COVID-19-positive patients with pneumonia were male patients with uncontrolled diabetes, hypertension, and obstructive and restrictive lung disease. COVID-19 is a pandemic disorder with a variable presentation and involves all the systemic disorders across all ages and genders. Pulmonary function is the most severely affected system and patients with extremes of age experience more severe disease. The exact mechanism is unknown, but patients with comorbidities such as hypertension, diabetes, and cardiac disorders have an increased risk of morbidity and mortality. COVID-19 has a variable presentation in different demographics, and most patients are symptomatic.
